# Genome-wide profiling reveals functional diversification of ∆FosB gene targets in the hippocampus of an Alzheimer's disease mouse model

**DOI:** 10.1371/journal.pone.0192508

**Published:** 2018-02-06

**Authors:** Jason C. You, Gabriel S. Stephens, Chia-Hsuan Fu, Xiaohong Zhang, Yin Liu, Jeannie Chin

**Affiliations:** 1 Department of Neuroscience and Farber Institute for Neurosciences, Thomas Jefferson University, Philadelphia, Pennsylvania, United States of America; 2 Memory & Brain Research Center, Department of Neuroscience, Baylor College of Medicine, Houston, Texas, United States of America; 3 Department of Neurobiology and Anatomy, University of Texas Medical School at Houston, Houston, Texas, United States of America; University of Modena and Reggio Emilia, ITALY

## Abstract

The activity-induced transcription factor ∆FosB has been implicated in Alzheimer’s disease (AD) as a critical regulator of hippocampal function and cognition downstream of seizures and network hyperexcitability. With its long half-life (> 1 week), ∆FosB is well-poised to modulate hippocampal gene expression over extended periods of time, enabling effects to persist even during seizure-free periods. However, the transcriptional mechanisms by which ∆FosB regulates hippocampal function are poorly understood due to lack of identified hippocampal gene targets. To identify putative ∆FosB gene targets, we employed high-throughput sequencing of genomic DNA bound to ∆FosB after chromatin immunoprecipitation (ChIP-sequencing). We compared ChIP-sequencing results from hippocampi of transgenic mice expressing mutant human amyloid precursor protein (APP) and nontransgenic (NTG) wild-type littermates. Surprisingly, only 52 ∆FosB gene targets were shared between NTG and APP mice; the vast majority of targets were unique to one genotype or the other. We also found a functional shift in the repertoire of ∆FosB gene targets between NTG and APP mice. A large number of targets in NTG mice are involved in neurodevelopment and/or cell morphogenesis, whereas in APP mice there is an enrichment of targets involved in regulation of membrane potential and neuronal excitability. RNA-sequencing and quantitative PCR experiments confirmed that expression of putative ∆FosB gene targets were altered in the hippocampus of APP mice. This study provides key insights into functional domains regulated by ∆FosB in the hippocampus, emphasizing remarkably different programs of gene regulation under physiological and pathological conditions.

## Introduction

The leucine zipper transcription factor ∆FosB belongs to the immediate early gene (IEG) family of proteins that are induced by neuronal activation to enact various activity-dependent programs in the cell [1]. ∆FosB is the truncated splice variant of FosB; this truncation is significant as it removes two C-terminal degron domains, making ∆FosB particularly resistant to proteasomal degradation [2]. Therefore, the half-life of ∆FosB is unusually long (~8 days *in vivo*), allowing it to modulate neuronal gene expression over prolonged periods of time [3]. The chronic and persistent nature of ∆FosB’s effect on neuronal and network function has been well-characterized in the nucleus accumbens, where it modifies addictive behavior following repetitive exposure to drugs of abuse [4]. Such studies also showed that ∆FosB epigenetically enhances or silences transcription of various target genes via recruitment of histone-modifying enzymes such as p300/CBP or HDAC1 to target gene promoters, respectively [5–7].

∆FosB expression is also increased in the hippocampus after seizure activity in various models of epilepsy where it has been used as a marker of activity [8–14]. Recently, we discovered that seizures and epileptiform activity also robustly increased ∆FosB expression in the hippocampus of a transgenic mouse model of Alzheimer’s disease (AD) expressing mutant human amyloid precursor protein (APP, line J20) as well as in pharmacological models of epilepsy [15, 16]. We found similar increases in ∆FosB expression in the hippocampus of AD patients and in patients with temporal lobe epilepsy [16]. The increase in ∆FosB expression had a significant impact on hippocampal function in APP mice, by suppressing expression of two genes essential for neuronal plasticity, *Fos* and *Calb1*, and by impairing hippocampus-dependent spatial memory [15, 16]. Treatment of APP mice with the antiepileptic drug levetiracetam reduced both epileptiform activity and ∆FosB expression, with concomitant improvement in cognition. Given evidence that seizures and epileptiform activity contribute to cognitive decline in AD [17, 18], the finding that seizures in AD increase hippocampal ∆FosB expression thus provides a mechanism to explain how even intermittent ictal events can cause persistent cognitive deficits.

However, considering the magnitude of ∆FosB upregulation following seizures, this unique transcription factor likely regulates many other aspects of hippocampal function in addition to neuronal plasticity. Consistent with this, viral overexpression of ∆FosB in area CA1 of the hippocampus altered dendritic spine morphology and modified behavior in several hippocampus-dependent tasks [19]. Therefore, to achieve a broader understanding of ∆FosB’s role in the hippocampus, we comprehensively profiled putative ∆FosB gene targets via high-throughput sequencing of genomic DNA bound by ∆FosB, enriched via chromatin immunoprecipitation (i.e. ∆FosB ChIP-sequencing, or ChIP-seq). Gene target profiles were generated using hippocampi obtained from both APP mice and nontransgenic (NTG) wild-type littermates from the same line of J20 APP mice used in our original studies [15, 16].

The results of this ChIP-seq analysis demonstrated that the repertoire of ∆FosB gene targets in the hippocampus covers broad functional domains, from cell-cell adhesion to regulation of ion channels and transporters. The ChIP-seq data also highlighted a diversification of ∆FosB’s functions under pathologic conditions, as both the number of gene targets and the number of over-represented gene ontology (GO) categories increased in APP versus NTG mice. Indeed, a comparison of major GO categories in NTG and APP mice revealed a significant functional shift in ∆FosB gene targets. In NTG mice, a large proportion of gene targets function in neurodevelopment and maintenance of neural circuitry. In contrast, targets involved in the regulation of membrane potential and neuronal excitability were highly represented in APP mice. Using RNA-seq and qPCR, we demonstrated that differential binding of ∆FosB to various gene targets in NTG and APP mice corresponds with downstream changes in mRNA expression. Together, our results highlight the diverse and complex programs of gene regulation that are coordinated by ∆FosB in the hippocampus of APP mice and potentially other animal models with recurrent seizures.

## Materials and methods

### Animals

Heterozygous APP transgenic mice expressing human APP carrying the Swedish (K670N, M671L) and Indiana (V717F) familial AD mutations (hAPP770 numbering) driven by the platelet-derived growth factor (PDGF) β chain promoter (Line J20) [20] were used in this study. The line was crossed for >10 generations onto a C57BL/6 background, and heterozygosity is maintained by breeding with wild-type C57BL/6 mice from The Jackson Laboratory. Male and female mice from this line that were 4 months of age were used for experiments. At this age, APP mice exhibit both recurrent seizures and cognitive deficits, but no plaque deposits [15, 20]. Age- and sex-matched nontransgenic (NTG) wild-type animals from the same line were used as controls. Mice were anesthetized with isoflurane and then transcardially perfused with saline. Brains were removed and hemisected: one hemibrain was post-fixed in 4% paraformaldehyde in phosphate-buffered saline for 48 hours at 4 degrees C, and the other hemibrain was flash- frozen on dry ice for biochemical experiments. All experiments involving animals were carried out in strict accordance with recommendations in the Guide for the Care and Use of Laboratory Animals of the National Institutes of Health, and followed protocol AN-6943 approved by the Institutional Animal Care and Use Committee of Baylor College of Medicine.

### Chromatin immunoprecipitation (ChIP)

We previously demonstrated that in the same line of APP mice used in this study, the frequency of seizures directly corresponds to the magnitude of ∆FosB expression in the dentate gyrus, and that treatment with the antiepileptic drug levetiracetam reduces epileptiform activity and normalizes ∆FosB expression [15, 16]. Notably, both epileptiform activity and the corresponding expression of ∆FosB is variable in this line of APP mice, as well as in other APP mice, and correlates inversely with cognitive function [15, 16]. Since the goal of the present experiments was to identify the gene targets bound by ∆FosB in APP mice with seizures and high levels of ∆FosB expression, we first confirmed that the APP mice used for ChIP-sequencing exhibit high ∆FosB expression. We did so by performing ΔFosB immunostaining on one hemibrain of each mouse in a group of 4-month old sex-matched APP and NTG mice (NTG, n = 15, 7 female and 8 male; APP, n = 15, 11 female and 4 male). ChIP was performed on the hippocampus of the opposite hemibrain from two female APP mice with high levels of ΔFosB expression and two female NTG controls, as previously described [15, 16]. Sonicated chromatin was processed using the Magna ChIP A Kit (Millipore). Antibodies were used at 2 μg/reaction for ChIP, and included rabbit anti-ΔFosB (Cell Signaling D3S8R) and normal rabbit IgG (Millipore 12–370). Prior to the addition of antibody, 2% of the sheared chromatin was set aside as input. Quality of ChIP DNA was assessed via qPCR analysis of *Calb1* gene pulldown by ΔFosB, which confirmed that ∆FosB bound *Calb1* in our ChIP samples, and that binding was increased in the two APP mice selected for ChIP-seq relative to NTG controls, as published previously [16]. Primers for *Calb1* gene amplification are as follows: 5'-TTCAAATACTCAACTGCCTCG-3' (forward); 5'-GGAGGCTTTCACTCCTGAATGT-3' (reverse).

### ChIP-sequencing (ChIP-seq) and peak mapping

ChIP-seq library preparation (NEBNext, New England Biolabs), sequencing, and peak calling was performed by the University of Pennsylvania Next-Generation Sequencing Core. Briefly, ΔFosB ChIP-seq enrichment data were obtained from Illumina hiSeq 2500 sequencing (single-read, 50-bp read depth). Statistically significant ChIP-seq peaks were identified by within-mouse comparison of regional read enrichment between ΔFosB ChIP and no-antibody input samples (negative control), mapped onto the *mm9* genome. A Fisher’s exact test with a Benjamini-Hochberg correction [21] calculated enrichment of ChIP signal (relative to input) for each identified peak, scoring by enrichment ratio and reporting the statistical significance of peaks with *p*<0.05. The gene closest to each statistically significantly enriched peak was then reported for each mouse, providing a list of putative genes bound by ΔFosB in both NTG and APP mice. Individual gene lists from animals of the same genotype were pooled to generate three lists: one for each genotype and a list containing genes with significantly enriched peaks shared by both genotypes.

### Gene ontology (GO) analysis

**Fig 1:**
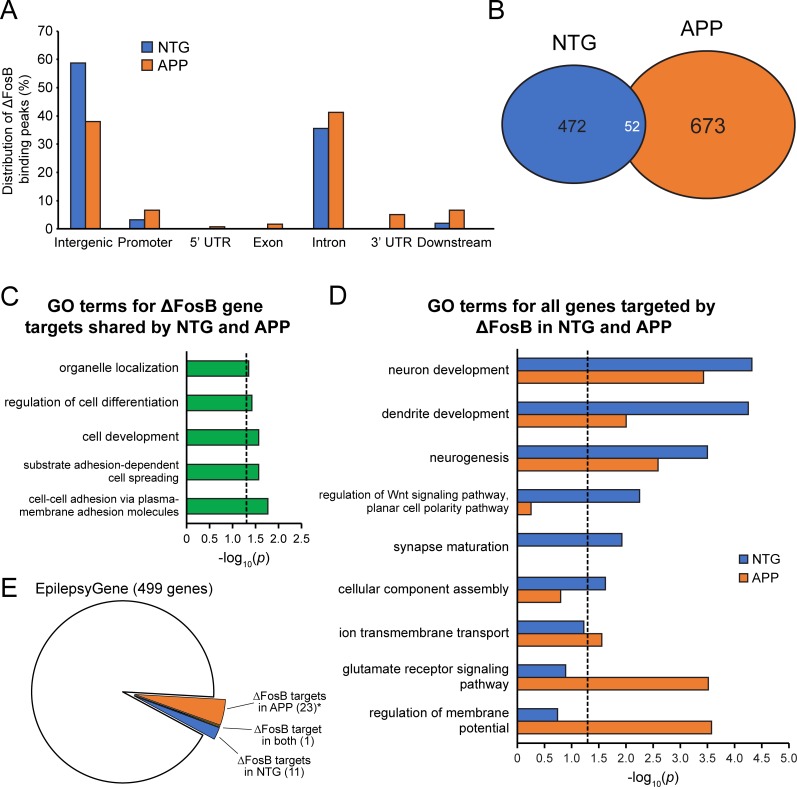
Increased binding of ΔFosB to gene regions in APP versus NTG mice corresponds with a functional shift in ΔFosB gene targets. *A*, Genomic distribution of ΔFosB binding peaks in NTG and APP mice. *B*, Number of genes targeted by ΔFosB in NTG and APP mice. *C*, Biological Process GO terms enriched by the 52 ΔFosB gene targets shared between NTG and APP mice. *D*, Biological Process GO terms enriched by all genes targeted by ΔFosB in NTG and APP mice. Dotted lines indicate p = 0.05 (two-sided hypergeometric tests with Benjamini-Hochberg correction). *E*, Schematic depicting the number of genes in the EpilepsyGene database that are also ∆FosB targets in NTG mice, APP mice, or both. *p = 0.039 using hypergeometric test for overlap between genes in the EpilepsyGene database and the list of ΔFosB target genes in APP mice.

Cytoscape 3.4.0 [22] was used in conjunction with the plug-in ClueGO 2.2.6 [23] to perform GO analysis. GO enrichment was determined for each genotype’s gene list using a two-sided hypergeometric test with a Benjamini-Hochberg correction, showing Biological Process GO terms significantly (*FDR*<0.05, GO updated 10/16/2016) enriched with genes from ChIP-seq-derived lists. In addition to calculating GO term enrichment, ClueGO was used to create functionally grouped annotation networks, showing significantly enriched GO terms as interconnected nodes with weighted lines (edges) that indicate the proportion of genes shared by different nodes. The color-coded clusters depicted for each genotype analysis are those containing the most highly significantly enriched GO terms from Fig 1, simplified to remove redundancy. Additional parameters were set to depict and highlight key aspects of each GO network. GO level minima were set to exclude overly broad GO terms from GO levels 1–2 and GO level maxima were set to return the maximal number of significant GO terms per gene list regardless of their level of detail. Kappa values were set iteratively to generate clusters of comparable inclusivity/size between the gene lists and other parameters were set to maximize information and visual clarity. Parameters for NTG network construction: GO level range = 3–16, kappa = 0.49, minimum number of genes included in term = 2, minimum percentage of genes included in term = 2%, GO term fusion/grouping = on. For APP network: GO level range = 3–15, kappa = 0.57, minimum number of genes included in term = 2, minimum percentage of genes included in term = 1.2%, GO term fusion/grouping = on.

### RNA extraction and qRT-PCR

RNA extraction was performed using adapted instructions from the Qiagen RNeasy Mini kit (74106). For these experiments, hippocampi were isolated from 2–3 month old mice (NTG, n = 17, 9 male and 8 female; APP, n = 13, 8 male and 5 female). Hippocampi were then submerged in RLT/β-mercaptoethanol buffer, minced with small scissors, and homogenized by passing the lysate through a 21G needle 15 times. Samples were centrifuged and the supernatants were transferred to new tubes. RNA was then purified according to the kit instructions, and eluted with nuclease-free water. Final RNA concentration was determined using a NanoDrop One spectrophotometer. Reverse transcription was performed using the TaqMan Reverse Transcription Reagent kit (ABI, N8080234) in accordance with the manufacturer’s instructions, also adding 2.5 μM random hexamers and oligo d(T)_16_ per reaction (ABI, N8080127 and N8080128). The resulting cDNA was diluted in water and used for quantitative PCR, which was performed with an ABI StepOnePlus machine using SYBR Green as a fluorophore. Each sample was run in triplicate reactions Primers used to amplify cDNA were as follows in forward (F) and reverse (R) format, each used at a concentration of 0.5 μM per reaction. *GAPDH*: F, 5’-AATTCAACGGCACAGTCAAGGC-3’ and R, 5’-TACTCAGCACCGGCCTCACC-3’; *Asef2*/*Spata13*: F, 5’-CTCAGAAGGCAGGACATG-3’ and R, 5’-CTCAGCCAAGGCAAAGAC-3’; *Gpc3*: F, 5′-GACTTGAAAGTGGAGACTGC-3′ and R, 5′-CCACATCCAGATCATAGGC-3′; *Magi1*: F, 5′-ACCACCAAACCAAAGCAG-3′ and R, 5′-AAGCGCAGAACATAAAGATC-3′; *Ano2* (GenBank: KC164761.2): F, 5′-GAGAGGAGCCTAGAAATCG-3′ and R, 5′-TGGTTGTCGTGAAAGTGC-3′.

### RNA-sequencing (RNA-seq)

Approximately 300 ng of RNA extracted from the dentate gyrus of APP mice with high ∆FosB expression and from wild-type NTG mice (4 per genotype; 3 male and 1 female each) were submitted to the University of Pennsylvania Next-Generation Sequencing Core, where library preparation and Illumina hiSeq 2500 sequencing (paired-end reads, 100-bp read depth) was performed. Raw mRNA reads were mapped to the mouse genome mm9. RNA-seq data were fit to a Gaussian distribution via quantile normalization and log_2_ transformation, and *p*-values for fold changes were corrected via false discovery rate (FDR). RNA-seq data were sex-compensated.

### Immunohistochemistry (IHC)

Tissue preparation and IHC were performed as previously described [15, 16]. Briefly, fixed brains were cryoprotected in 30% sucrose prior to sectioning at 30 μm thickness, and sections were stored in cryoprotectant (30% glycerol, 30% ethylene-glycol in PBS) until processing. Ten subseries were collected throughout the rostral-caudal extent of the brain. For staining with 3,3’-diaminobenzidine (DAB, Sigma), the antibodies used were rabbit anti-ΔFosB (Cell Signaling) and biotinylated goat anti-rabbit secondary (Vector). Further amplification was accomplished using Avidin-Biotin Complex (Vectastain) application. ΔFosB-IR quantification was performed by assessing the mean pixel intensity using ImageJ software by a genotype-blinded observer.

### Statistics

General statistical analyses were performed using SPSS-23 (IBM), Prism 7 (GraphPad), and R [24]. Sample sizes for both biochemical and behavioral experiments were determined based on calculations performed on accumulated empirical data and power analyses. The number of animals used for each experiment was appropriate to detect biochemical or behavioral differences. Unless otherwise stated, results are represented as sample means ± standard errors of the mean. These data are distributed normally as stipulated by the central limit theorem. Differences between experimental groups were assessed by unpaired, two-tailed Student’s t test when comparing means between two groups. Correlations were assessed by simple regression analysis. No specific method of randomization was used, but animals were semi-randomly assigned to experimental groups based on birth order after balancing for age, sex, and genotype. Hypergeometric analyses were performed to test the statistical significance of the overlap between two lists of genes using R.

## Results

### Increased binding of ΔFosB to gene regions in APP versus NTG mice

High-throughput sequencing and genomic mapping of ΔFosB-bound DNA identified 2,343 significant binding peaks for ΔFosB in the hippocampus of NTG and APP mice. Subsequent gene association analysis with Hypergeometric Optimization of Motif EnRichment (HOMER) [25] found 1,197 total genes that were putative hippocampal ΔFosB targets in NTG and/or APP mice. Notably, we detected a substantial shift in genomic regions containing ΔFosB binding peaks between NTG and APP mice (Fig 1A). A large proportion of ΔFosB binding peaks in NTG mice occurred in intergenic regions (58.81%) and introns (35.59%), which may be related to the high number of accessible chromatin sites in these genomic regions [26]. However, in APP mice we observed a redistribution of ΔFosB binding peaks, as peaks shifted away from intergenic regions (decreased from 58.81% in NTG mice to 38.09% in APP mice) to bind various regions associated with genes. Regions that exhibited enrichment of ΔFosB binding peaks in APP versus NTG mice included gene promoter, 5’ UTR, exon, intron, 3’ UTR regions. Consistent with the increased binding of ΔFosB to gene-associated regions in APP mice, we found that the number of total ΔFosB gene targets increased from 524 in NTG mice to 725 in APP mice (Fig 1B).

### ∆FosB gene targets function in distinct biological pathways in NTG and APP mice

Interestingly, we found that out of the 1197 total putative hippocampal ∆FosB gene targets, only 52 genes (~4%) were shared by NTG and APP mice (Fig 1B and S1 Table); the vast majority of targets appeared to be unique either to NTG (472 genes, 39.4%) or APP mice (673 genes, 56.2%). Given the low number of genes similarly bound by ∆FosB in NTG and APP mice, we hypothesized that ∆FosB may perform disparate functions in NTG and APP mice by differentially regulating genes belonging to distinct molecular/cellular pathways. Therefore, to characterize the kinds of genes targeted by ∆FosB under physiologic and pathologic conditions, we used Cytoscape [22] in combination with the plug-in ClueGO [23] to perform a GO analysis of ∆FosB gene targets. This analysis categorizes gene targets by biological function and groups genes that operate within the same molecular/cellular pathway. Each gene group is associated with a GO term that describes the biological function performed by genes of that group, and a *p-*value that indicates the significance of this particular GO term enrichment.

We first assessed GO terms for the 52 ∆FosB gene targets shared by NTG and APP mice. The GO terms significantly enriched by these shared genes corresponded with fundamental cellular processes, such as organelle localization, cell development, differentiation, and cell-cell adhesion (Fig 1C). We then evaluated major GO terms belonging to all 1197 genes targeted by ∆FosB in NTG and APP mice (Fig 1D). We found that despite the fact that ∆FosB largely targeted different genes in NTG and APP mice, there was significant enrichment of GO terms for neuron development, dendrite development, and neurogenesis in both types of mice. However, we also found that these three GO terms were consistently less enriched in APP mice than in NTG mice, suggesting that ∆FosB in APP mice also targeted many genes that do not function in these domains. Further analysis of major GO terms in APP mice revealed that ∆FosB targeted a significant number of genes involved in ion transmembrane transport, glutamate receptor signaling, and regulation of membrane potential. Notably, those GO terms were not significantly enriched in NTG mice. In contrast, GO terms related to Wnt signaling, synapse maturation, and cellular component assembly were significantly enriched in NTG mice, but not APP mice.

The diversification of GO terms in APP mice to include ion transport, glutamate receptor signaling, and regulation of membrane potential was striking given the recurrent seizures exhibited by both AD patients and mouse models, as well as the critical role that seizures play in cognitive deficits [15, 16, 27–29]. We therefore assessed whether ∆FosB gene targets in APP mice are involved in epilepsy-related pathways by comparing our list of gene targets to EpilepsyGene, a database cataloging 499 genes and 3931 gene variants that have been implicated in clinical epilepsy [30]. Hypergeometric enrichment analyses revealed that genes bound by ∆FosB in APP mice were significantly overrepresented in the EpilepsyGene list (p = 0.039), whereas genes bound by ∆FosB in NTG mice were not (p = 0.52) (Fig 1E). Table 1 lists the ∆FosB target genes in APP mice that are also represented in the EpilepsyGene database. Notably, only 1 gene (*braf*) in Table 1 was bound by ∆FosB in both APP and NTG mice; all others were uniquely bound in APP mice.

**Table 1:** ∆FosB targets in APP mice that are included in the EpilepsyGene database.

Gene	Gene name
*abcc8*	ATP binding cassette subfamily C member 8
*ank3*	Ankyrin-3; Ankyrin-G
*braf*	B-Raf transforming gene
*chd2*	Chromodomain helicase DNA-binding protein 2
*cntn5*	Contactin 5
*cntnap2*	Contactin-associated protein-like 2
*gpr98*	Adhesion G protein-coupled receptor V1
*heg1*	Heart development protein with EGF-like domains
*kcnd2*	Potassium voltage-gated channel, Shal-related family member 2; Kv4.2
*kcnh5*	Potassium voltage-gated channel subfamily H member 5; Eag2
*lama2*	Laminin subunit alpha 2
*mef2c*	Myocyte enhancer factor 2C
*n6amt1*	N-6 adenine-specific DNA methyltransferase 1
*nedd4l*	Neural precursor cell expressed, developmentally down-regulated 4-like, E3 ubiquitin protein ligase
*oprm1*	Opioid receptor mu 1
*pacs2*	Phosphofurin acidic cluster sorting protein 1
*pcdh19*	Protocadherin 19
*pign*	Phosphatidylinositol glycan anchor biosynthesis class N protein
*ptprr*	Protein tyrosine phosphatase, receptor type R
*scn9a*	Sodium channel voltage-gated type IX, alpha; Nav1.7
*slc35a3*	Solute carrier family 35 (UDP-N-acetylglucosamine (UDP-GlcNAc) transporter), member 3
*ss18l1*	SS18; nBAF chromatin remodeling complex subunit like 1
*wnk1*	WNK lysine deficient protein kinase 1

### GO network analysis reveals expanded and diversified repertoire of ∆FosB targets in APP mice

**Fig 2:**
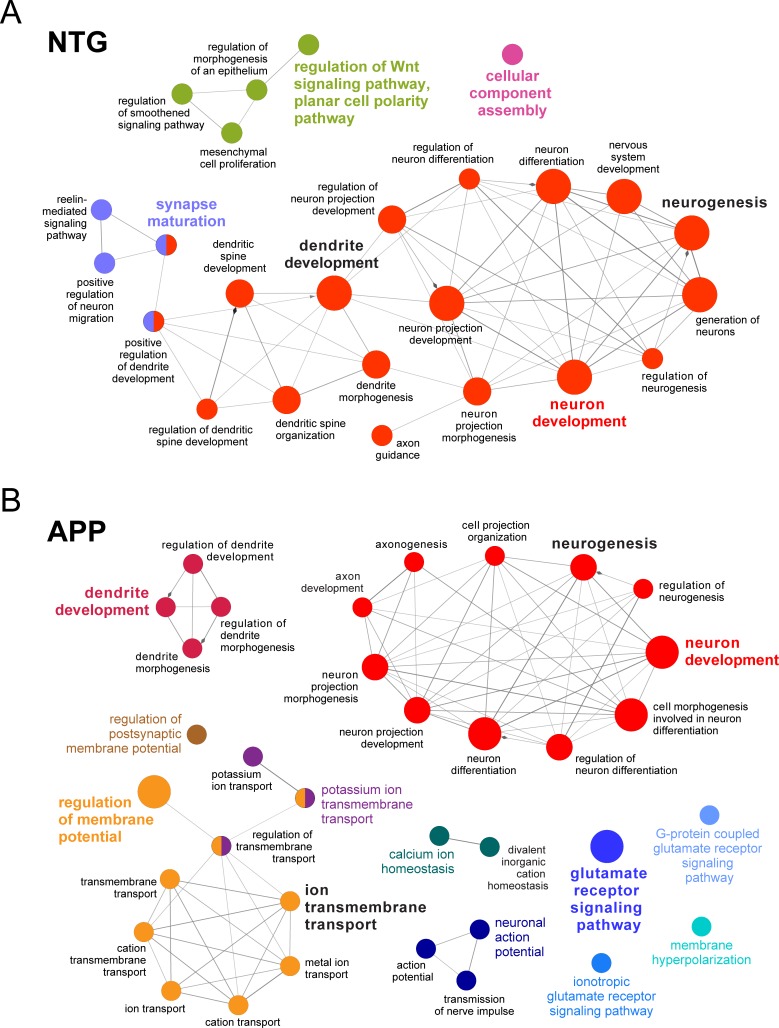
Expansion and diversification of GO terms enriched by ΔFosB gene targets in APP versus NTG mice. Nodal network diagrams showing clusters of significant GO terms for NTG (*A*) and APP (*B*) mice. Node size inversely corresponds with *p*-values from two-sided hypergeometric tests with Benjamini-Hochberg correction. Weighting of lines between GO term nodes denotes proportion of shared genes between nodes.

To gain more comprehensive insight into the repertoire of genes targeted by ∆FosB in NTG and APP mice, we performed a nodal network analysis of GO terms enriched by ∆FosB gene targets in these mice (Fig 2). Since many genes are known to perform multiple cellular functions, different gene groups can contain similar genes. By connecting ∆FosB-enriched gene groups via shared genes, coordinated gene networks that are simultaneously targeted by ∆FosB in NTG and APP mice can be assessed. This network-level analysis of ∆FosB gene targets provides a comprehensive view of distinct cellular programs engaged by ∆FosB under physiologic and pathologic conditions. Consistent with our assessment of individual GO terms in Fig 1D, nodal network analysis of enriched GO clusters in NTG mice reveal an extensively interconnected cluster containing GO terms neuron development, dendrite development, neurogenesis, and synapse maturation, as well as a Wnt signaling pathway network involving the regulation of cell polarity (Fig 2A). Overall, the GO network of NTG mice highlights involvement of ∆FosB in pathways critical for neurodevelopment and maintenance of the neural network architecture.

By comparison, the GO network of APP mice shows an expanded and diversified array of GO terms, consistent with the expanded and diversified array of ∆FosB gene targets in APP mice (Fig 2B). Unlike the nodal network diagram of NTG mice, the APP diagram displays numerous clusters containing GO terms enriched with genes related to neuronal excitability, glutamatergic neurotransmission, and action potential regulation. Notable GO terms and clusters in APP mice that did not appear in NTG mice contain genes for 1) ion channels, ion transporters, and channel-localizing scaffolding proteins, and 2) genes linked to calcium homeostasis. Although GO clusters relating to neurodevelopmental processes are represented in APP mice, these clusters contain fewer GO terms and are less interconnected than their NTG counterparts. These results demonstrate a functional shift of ∆FosB gene targets away from neurodevelopmental processes and towards regulation of excitability in APP mice.

### Binding of ∆FosB to putative targets corresponds to changes in gene transcription

Given the crucial role of ∆FosB in epigenetic gene regulation [31], differential binding of ∆FosB to gene targets in APP and in NTG mice genotypes is likely to have a major effect on neuronal gene expression. Therefore, to evaluate whether alterations in the binding of ∆FosB to various gene targets correspond with downstream changes in transcription, we analyzed the mRNA expression of four representative ∆FosB gene targets that demonstrated differential ∆FosB binding in NTG versus APP mice. These genes were selected from major representative GO clusters in NTG and APP mice.

**Fig 3:**
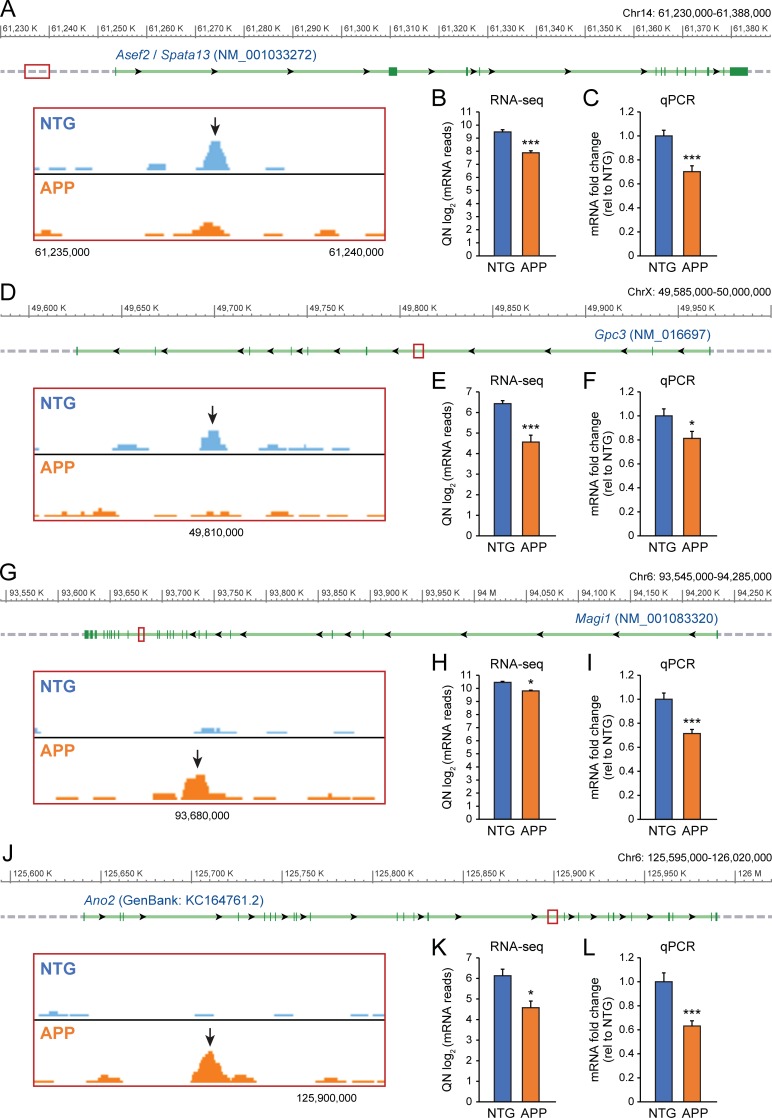
Differential binding of ΔFosB to target genes in NTG and APP mice corresponds with changes in downstream mRNA expression. *A*,*D*,*G*,*J*, NCBI genome browser visualization of the *Asef2* (*Spata13)*, *Gpc3*, *Magi1*, and *Ano2* loci. Locations of ΔFosB ChIP-seq binding peaks are marked on gene tracks by red boxes, with respective insets displaying magnified peaks (arrows). *B*,*C*, *Asef2 (Spata13)* mRNA expression in NTG and APP mice, quantified via RNA-seq (***p = 1.3x10^-9^) and qPCR (***p = 1.7x10^-4^). *E*,*F*, *Gpc3* mRNA expression in NTG and APP mice, quantified via RNA-seq (***p = 1.2x10^-4^) and qPCR (*p = 0.035). *H*,*I*, *Magi1* mRNA expression in NTG and APP mice, quantified via RNA-seq (*p = 0.02) and qPCR (***p = 2x10^-4^). *K*,*L*, *Ano2* mRNA expression in NTG and APP mice, quantified via RNA-seq (*p = 0.028) and qPCR (***p = 1x10^-3^). For RNA seq n = 4 per genotype, for qPCR n = 13–17 per genotype. Differences between genotypes were assessed using Benjamini-Hochberg FDR for RNA-seq data, and unpaired Student’s t-test for qPCR data. QN, quantile normalization.

We first analyzed mRNA expression of *Asef2* (also known as *Spata13*) and *Gpc3*, two genes that ChIP-seq binding peak analysis indicated were significantly bound by ∆FosB in NTG mice, but not APP mice (Fig 3A and 3D). These genes belong to the neurodevelopmental GO cluster (2A, red/blue) of NTG mice. *Asef2* encodes a guanine nucleotide exchange factor that regulates the actin cytoskeleton [32, 33] and facilitates dendritic spine and synapse formation [34]. *Gpc3* encodes glypican 3, a heparan sulfate proteoglycan (HSPG) implicated in hippocampal patterning and excitatory synapse development [35–37]. Using unbiased RNA-seq and qPCR analyses, we found that differential binding of ∆FosB to these genes in NTG and APP mice was indeed associated with downstream changes in mRNA expression. Our data showed that mRNA levels of both *Asef2* (Fig 3B and 3C) and *Gpc3* (Fig 3E and 3F) were decreased in APP versus NTG mice, coinciding with decreased ∆FosB binding to these genes.

We also analyzed the mRNA expression of *Magi1* and *Ano2*, genes that were significantly bound by ∆FosB in APP mice, but not NTG mice (Fig 3G and 3J). These genes were selected from the APP GO cluster containing ion transmembrane transport and regulation of membrane potential. *Magi1* encodes a scaffolding protein that regulates the calcium-activated big potassium (BK) channel [38, 39]. *Ano2* encodes anoctamin 2, a somatodendritic calcium-activated chloride channel [40–42]. Differential binding of ∆FosB to *Magi1* and *Ano2* was also associated with changes in mRNA expression, as increased binding of ∆FosB to these genes in APP mice corresponded with decreased mRNA expression of both *Magi1* (Fig 3H and 3I) and *Ano2* (Fig 3K and 3L).

Of the four example genes described here, ∆FosB bound to an intergenic region upstream of *Asef2*, and to intronic regions of *Gpc3*, *Magi1* and *Ano2* (see Fig 3). We also assessed the distribution of genomic regions bound by ∆FosB for other genes that exhibited differential expression in RNA-seq (S1 Fig), and examined whether binding was associated with increased or decreased gene expression. All ∆FosB target genes that were differentially regulated in RNA-seq were bound in either promoter, intergenic, or intronic regions. Notably, gene expression could be increased or decreased in association with binding of ∆FosB to any of these genomic regions. This finding suggests that the type of genomic region bound by ∆FosB is unlikely to be a determinant of the direction of gene expression modulation.

## Discussion

We have generated a comprehensive ChIP-seq profile of ∆FosB gene targets in the hippocampus of both NTG and APP mice. The characterization of ∆FosB gene targets via GO analysis also highlight two apparently distinct programs of hippocampal gene regulation under physiologic and AD-associated pathologic conditions. We found that in the normal hippocampus of NTG mice, ∆FosB targeted many genes involved in neuronal development and cell morphogenesis, implicating its role in the formation and maintenance of neural circuitry. The putative role of ∆FosB in dendrite and synapse development is particularly interesting considering that hippocampal expression of activity-dependent ∆FosB is sparse under normal circumstances [10, 13–16]. We thus hypothesize that expression of ∆FosB under physiologic conditions may serve to modulate synaptic plasticity in a small population of highly active hippocampal neurons, perhaps as a homeostatic mechanism. Consistent with our findings, a recent study found that spatial learning and novel environment exposure induces hippocampal expression of ∆FosB, which plays a crucial role in regulating dendritic spine morphology [19]. Furthermore, homeostatic control of neuronal plasticity is a known function of many other IEG proteins [1, 43, 44].

However, in the pathologic hippocampus of APP mice, we found that the repertoire of ∆FosB gene targets expanded and diversified to include genes involved in the regulation of membrane potential and neuronal excitability. This finding suggests a functional shift in ∆FosB gene targets that occurs with seizure-induced ∆FosB accumulation. This functional shift is significant considering the aberrant hyperexcitability of the APP hippocampus [45–47]. Since robustly increased expression of ∆FosB in the APP hippocampus is a direct result of neuronal hyperactivity [16], the putative shift in ∆FosB function towards regulation of neuronal excitability may in turn represent a compensatory neuroprotective response to limit excitotoxicity. Consistent with this view, a recent study found that ∆FosB could protect neurons from ischemic injury [48]. We have also shown in previous studies that ∆FosB impairs hippocampal function and cognition [15, 16], likely by reducing synaptic plasticity and neuronal response to stimulation. Together, these findings suggest that ∆FosB regulates a critical balance between neuronal function and neuroprotection in the hyperexcitable hippocampal network of APP mice.

We also assessed how changes in the binding of ∆FosB to various targets in NTG and APP mice could impact downstream transcription. Via RNA-seq and qPCR analyses, we showed that genes exhibiting both increased and decreased ∆FosB binding in APP mice also demonstrated alterations in mRNA expression. Interestingly, the four ∆FosB gene targets we investigated all had decreased mRNA expression in APP relative to NTG mice, despite opposite trends in ∆FosB gene binding. *Asef2* and *Gpc3* had high ∆FosB binding in NTG mice, whereas *Magi1* and *Ano2* had high ∆FosB binding in APP mice. Together, these results suggest that ∆FosB promotes transcription of *Asef2* and *Gpc3* in NTG mice, and suppresses *Magi1* and *Ano2* transcription in APP mice. These results are consistent with previous studies showing that ∆FosB has the ability to bidirectionally alter gene expression [4]. Decreased mRNA expression of *Magi1* in APP mice is also notable considering that Magi1 sequesters BK channels from cell surfaces [39]. Reduction of Magi1 protein in the APP hippocampus would allow increased surface expression of BK channels, which in turn could increase potassium conductance and reduce neuronal excitability. In addition, ∆FosB-mediated suppression of *Ano2* transcription in the hippocampus could also reduce neuronal excitability since Ano2 affects membrane repolarization, and mice in which *Ano2* has been ablated exhibit decreased excitability of inferior olive neurons [40–42]. Therefore, validation of ∆FosB-mediated suppression of *Magi1* or *Ano2* could provide further support for a neuroprotective role of ∆FosB in APP mice.

Seizure-induced accumulation of ∆FosB likely contributes to the expansion and functional diversification of ∆FosB gene targets in APP mice. However, differences in the chromatin accessibility landscape of APP versus NTG neurons may also have a major impact on how ∆FosB interacts with the genome. Recent studies found that neuronal activity or behavioral training cause significant chromatin reorganization in hippocampal neurons [26, 49]. Activity appears to largely induce chromatin relaxation at gene regions throughout the genome, allowing various IEG transcription factors such as cFos and FosB to access gene targets [26]. Considering that ∆FosB is closely related to FosB, these activity-induced changes in chromatin structure likely affect accessibility of gene targets to ∆FosB as well, and may contribute to the increased number and diversity of genes bound by ∆FosB in APP versus NTG mice. Indeed, neuronal hyperexcitability in the APP hippocampus may chronically alter chromatin structure in a way that allows ∆FosB to bind genes that are normally inaccessible under physiologic conditions. Consistent with this view, the binding peak distribution of ∆FosB in APP mice aligns closely with genomic regions found to exhibit chromatin relaxation after neuronal activation [26]. In conclusion, our findings have provided critical insight into the diverse functions of ∆FosB in the hippocampus under both physiologic and pathologic conditions. Future characterization of how ∆FosB influences hippocampal gene regulation may aid the discovery of novel therapeutic targets to improve cognition and neuroprotection in AD and other seizure-related disorders.

## Supporting information

S1 Fig Binding of ∆FosB to specific genomic regions is associated with both increased and decreased gene expression. Genes bound by ΔFosB in ChIP-seq that showed differential expression at the p = 0.1 level by RNA-seq were subdivided by the genomic regions at which they were bound. Numbers within bars denote the percentage of genes that were increased or decreased for each region and genotype.
(EPS)

S1 Table ∆FosB gene targets in APP mice, NTG mice, or both. ∆FosB ChIP-seq enrichment data were analyzed, as described in the Methods, to generate lists of genes putatively bound by ∆FosB in either APP mice, NTG mice, or in both genotypes. Lists of genes for each genotype, or shared between the genotypes, can be found on individual tabs within the file.
(XLSX)
